# Efficacy of conservative treatment for spastic cerebral palsy children with equinus gait: a systematic review and meta-analysis

**DOI:** 10.1186/s13018-022-03301-3

**Published:** 2022-09-08

**Authors:** Krongkaew Klaewkasikum, Tanyaporn Patathong, Patarawan Woratanarat, Thira Woratanarat, Kunlawat Thadanipon, Sasivimol Rattanasiri, Ammarin Thakkinstian

**Affiliations:** 1 Department of Orthopaedics, Faculty of Medicine Ramathibodi Hospital, Mahidol University, Bangkok, 10400 Thailand; 2 Department of Preventive and Social Medicine, Faculty of Medicine, Chulalongkorn University, Bangkok, 10330 Thailand; 3 Department of Clinical Epidemiology and Biostatistics, Faculty of Medicine Ramathibodi Hospital, Mahidol University, Bangkok, 10400 Thailand

**Keywords:** Ankle, Equinus, Cerebral palsy, Gait, Management

## Abstract

**Background:**

Comparisons between various conservative managements of spastic equinus deformity in cerebral palsy demonstrated limited evidences, to evaluate the efficacy of conservative treatment among cerebral palsy children with spastic equinus foot regarding gait and ankle motion.

**Methods:**

Studies were identified from PubMed and Scopus up to February 2022. Inclusion criteria were randomized controlled trial (RCT), conducted in spastic cerebral palsy children with equinus deformity, aged less than 18 years, compared any conservative treatments (Botulinum toxin A; BoNT-A, casting, physical therapy, and orthosis), and evaluated gait improvement (Physician Rating Scale or Video Gait Analysis), Observational Gait Scale, Clinical Gait Assessment Score, ankle dorsiflexion (ankle dorsiflexion at initial contact, and passive ankle dorsiflexion), or Gross Motor Function Measure. Any study with the participants who recently underwent surgery or received BoNT-A or insufficient data was excluded. Two authors were independently selected and extracted data. Risk of bias was assessed using a revised Cochrane risk-of-bias tool for randomized trials. I^2^ was performed to evaluate heterogeneity. Risk ratio (RR), the unstandardized mean difference (USMD), and the standardized mean difference were used to estimate treatment effects with 95% confidence interval (CI).

**Results:**

From 20 included studies (716 children), 15 RCTs were eligible for meta-analysis (35% had low risk of bias). BoNT-A had higher number of gait improvements than placebo (RR 2.64, 95% CI 1.71, 4.07, *I*^2^ = 0). Its combination with physical therapy yielded better passive ankle dorsiflexion at knee extension than physical therapy alone (USMD = 4.16 degrees; 95% CI 1.54, 6.78, *I*^2^ = 36%). Casting with or without BoNT-A had no different gait improvement and ankle dorsiflexion at knee extension when compared to BoNT-A. Orthosis significantly increased ankle dorsiflexion at initial contact comparing to control (USMD 10.22 degrees, 95 CI% 5.13, 15.31, *I*^2^ = 87%).

**Conclusion:**

BoNT-A and casting contribute to gait improvement and ankle dorsiflexion at knee extension. BoNT-A specifically provided gait improvement over the placebo and additive effect to physical therapy for passive ankle dorsiflexion. Orthosis would be useful for ankle dorsiflexion at initial contact.

*Trial registration* PROSPERO number CRD42019146373.

**Supplementary Information:**

The online version contains supplementary material available at 10.1186/s13018-022-03301-3.

## Background

Equinus is a common foot deformity in spastic cerebral palsy patients with 83.3% prevalence [1]. The concerning problems include toe walking [2], foot pain, plantar fasciitis [3, 4], gait instability, impaired oxygen uptake rate, walking intolerance, and inability for long-distance walking [5]. The equinus is defined as the dynamic or static ankle plantar flexion position that may preclude plantigrade foot [6]. Ankle passive range of motion and gait assessment using the initial score for foot contact by Physician Rating Scale (PRS) are basically used for equinus evaluation [7]. Gait assessment comprised of the instrumented 3-dimensional and the observational gait analysis [8]. The instrumented gait analysis is the gold standard for classifying equinus (ankle dorsiflexion at initial contact) in cerebral palsy [8]. The observational gait assessments for equinus foot (Table 1) were PRS based on gait pattern, hindfoot, and ankle position at foot contact [7, 9, 10]; Video Gait Analysis (VGA) graded initial foot contact [11, 12]; Observational Gait Scale focused on initial foot contact, foot contact mid-stance, heel rise, and hindfoot [8]; and Clinical Gait Assessment Score (CGAS) evaluated foot at initial contact, stance phase, and terminal stance [13]. The Gross Motor Function Measure (GMFM), specifically for dimension D: standing, and E: walking, running, and jumping, is also widely applied for monitoring and tailoring equinus treatment to optimize the rehabilitation for cerebral palsy children [14]. Prolonged equinus may lead to fixed deformity which requires surgical treatment. Hence, early management is mandatory to minimize progression and encourage gait efficiency [15].

**Table 1:** The observational gait assessments for equinus foot

Gait assessment scales	Subscales/sections	Number of items	Total score
Physician’s Rating Scale (PRS) [7]	Crouch, equinus gait, hindfoot, knee, speed of gait, gait	6	28 (14 points/limb)
Modified PRS [9]	Crouch, knee, foot contact, change	4	20 (10 points/limb)
Abbreviated PRS [10]	Crouch, foot contact	2	14 (7 points/limb)
Video Gait Analysis (VGA) [11]	Initial foot contact (graded as flatfoot, toe then heel, mild toe walking, marked toe walking)	1	8 (4 points/limb)
Modified VGA [12]	Initial foot contact (graded as heel–toe, foot–flat, toe–toe)	1	6 (3 points/limb)
Observational Gait Scale [8]	Knee mid-stance, initial foot contact, foot contact mid-stance, heel rise, hindfoot, base of support, assistive devices, change	8	44 (22 points/limb)
Clinical Gait Assessment Score (CGAS) [13]	Swing, initial contact, stance phase, terminal stance	4 (14 body parts)	96 (48 points/limb)

For dynamic equinus deformity in spastic cerebral palsy children, botulinum toxin A (BoNT-A), casting, orthosis, and physiotherapy are recommended [16]. BoNT-A can improve gait pattern measured by PRS and VGA with minor side effects when compared to placebo [17]. BoNT-A plus delayed casting might be the best treatment to improve ankle dorsiflexion at stance, while BoNT-A alone was at the highest rank for passive ankle motion at knee extension, followed by immediate casting, BoNT-A plus delayed casting, and BoNT-A with immediate casting [18]. However, none of them showed significantly a different peak dorsiflexion at stance and passive ankle motion [18]. The posterior ankle–foot orthosis (AFO) significantly increased ankle dorsiflexion at initial contact in children with equinus gait when compared to bare foot [19]. Physiotherapy, i.e., stretching technique and strengthening, is commonly performed in adjunct with other treatments [13, 20, 21].

With regard to BoNT-A, casting, AFO, and physiotherapy for equinus treatment, the previous systematic reviews and meta-analysis were limited due to various casting protocols [18], improper effect size estimation [17], no risk-of-bias assessment [17, 19], and no comparisons among these conservative treatments. Therefore, we aimed to conduct a systematic review and meta-analysis comparing usual care/placebo/control, BoNT-A, casting, physiotherapy, and orthosis in terms of the changes in gait and ankle movement in spastic cerebral palsy children presenting with equinus foot.

## Methods

This study was conducted in accordance with the Preferred Reporting Items for Systematic Reviews and Meta-Analyses (PRISMA) guidelines [22]. The research was registered on PROSPERO and can be accessed online (PROSPERO number CRD42019146373).

### Search strategy

PubMed and Scopus databases were systematically searched up to February 2022. The searching terms were ‘cerebral palsy’ AND (‘botulinum*’ OR ‘BTX’ OR ‘BoNT-A’ OR ‘Botox’ OR ‘Dysport’ OR ‘cast’ OR ‘casts’ OR ‘casting’ OR ‘physical therapy’ OR ‘physiotherapy’ OR ‘orthotic’ OR ‘splint’ OR ‘bracing’ OR ‘brace’).We also included reference lists of selected articles and previous meta-analysis articles without limitation of language. The details of searching strategy are provided in Additional file 1.

### Study selection

The inclusion criteria of the studies were randomized controlled trial (RCT), conducted in children aged less than 18 years with spastic cerebral palsy, equinus deformity (i.e., an equinus foot positioning during the stance phase of the gait, or passive ankle dorsiflexion with knee extension < 10 degrees), Gross Motor Function Classification System (GMFCS) level I-III, compared the effect of any conservative treatments (i.e., placebo, BoNT-A, casting, physical therapy, orthosis, and/or combination of those treatments), and evaluated the effect of conservative treatment on gait (number of gait improvement, composite score of gait assessment, ankle dorsiflexion at stance of gait cycle), or range of ankle motion (passive ankle dorsiflexion with knee extension) or GMFM dimension D (standing) and E (walking, running, and jumping). Reasons why the studies were ineligible were documented in the PRISMA flow diagram as records removed by search filters.

The exclusion criteria were the eligible or included studies with the participants who underwent surgery less than 12 months or received BoNT-A less than 6 months or insufficient data for pooling after three attempts to contact the authors.

The title and abstract screening was independently performed by two authors (KK and TP). Subsequently, the full text of selected articles was retrieved and reviewed by two authors. Any conflicts were adjudicated by group discussion with all authors.

### Data extraction

Data were independently extracted by two authors (KK and TP) using a standardized data extraction form with the following information: author(s), year of publication, study design, sample size, participant’s characteristics (gender, age, type of cerebral palsy, level of GMFCS, intervention, time of follow-up, intensity–dose of intervention, duration of intervention), and outcomes (type of data, measurement). The outcomes were the number of gait improvement measured by PRS and VGA, the composite score of gait assessed by PRS, Observational Gait Scale, or CGAS, ankle dorsiflexion at initial contact from 3-dimensional gait analysis, passive range of ankle dorsiflexion, and GMFM dimensions D and E. The number of gait improvement at least 2 points of total PRS score (either PRS 6 subscales, total 14 points/limb [7, 20, 23], modified PRS 4 subscales, total 10 points/limb [9], or abbreviated PRS 2 subscales, total 7 points/limb [10]) or at least 1 point of total VGA score (either VGA 4 grades, total 4 points/limb [11], or modified VGA 3 grades, total 3 points/limb [12]) from the baseline was clinically significant and categorized as improve [11, 23]. Composite scores were calculated by combining subscale scores of each observational gait assessment into total scores. Since there were various observational gait assessments with their modifications, subscales/sections and total scores were individually detailed. Composite scores of PRS were reported as 6 subscales, total 14 points/limb [7, 20, 23], 4 subscales, total 10 points/limb [9], and 2 subscales, total 7 points/limb [10]. Composite scores of Observational Gait Scale involved 8 sections, total 22 points/limb, and those of CGAS contained 4 phases of gait, total 48 points/limb [13]. Passive ankle dorsiflexion was generally reported with knee extension. Dichotomous outcome was identified from included studies as risk ratio with 95% confidence interval (CI) for number of gait improvement. Continuous outcomes were extracted as mean, median, and standard deviation for the composite score of gait scale, degrees of ankle dorsiflexion at initial contact, passive range of ankle dorsiflexion and GMFM. Any disagreements between the two reviewers were resolved by group discussion.

### Quality assessment of the reviewed studies

A revised Cochrane risk-of-bias tool for randomized trials (RoB 2) was used to assess the risk of bias [24]. The risk of bias was rated as 'Low' or 'High' risk of bias, or labeled as 'Some concerns'. Two authors (KK and TP) independently rated methodological quality of the studies. Any discrepancies were discussed till achieving final conclusion.

### Statistical analysis

Meta-analyses were performed using Stata program version 15 (Statcorp, College Station, TX, USA). Treatment effects were calculated using RR and 95%CI for the dichotomous outcome, and post-intervention mean and standard deviation (SD) for continuous outcomes. The dichotomous number of gait improvement was presented as pooled RR with 95%CI. RR equal to 1 indicated the same treatment effects between groups. RR < 1 means the treatment is less likely to have gait improvement, and RR > 1 means the treatment is more likely to have gait improvement when compared to the reference group. The standardized mean difference (SMD) with 95%CI was estimated according to different composite scores of gait improvement. SMD is the standard method used for pooling difference scales in the meta-analysis. It was calculated based on mean difference divided by standard deviation and reported as Cohen’s d. SMD was interpreted according to Cohen’s d as 0 for no effect, 0.2 for small, 0.5 for medium, and 0.8 for large effect. SMD < 0 means the treatment is less efficacious, and SMD > 0 means the treatment is more efficacious than the comparison group. The unstandardized mean difference (USMD) with 95%CI was analyzed for degrees of ankle dorsiflexion at initial contact and passive range of ankle dorsiflexion which were used the same scale. USMD equal to zero represented indifference between treatment pairs. USMD < 0 means the treatment has less effect, and USMD > 0 means the treatment has more effect than the comparison group. A fixed-effect model by inverse-variance method was performed if treatment effects between studies were homogeneity (p value of Cochrane Q statistics > 0.1 or *I*^2^ test < 25%); otherwise, a random effect model using the DerSimonian and Laird method was applied [25]. Source of heterogeneity was explored according to the characteristics of studies and interventions, i.e., subgroup analysis. Sensitivity analysis was considered for specific factors contributed to the outcomes. Funnel plot and Egger’s test were assessed for a publication bias [26]. When the corresponding p value of Egger’s test was less than 0.05, a contour-enhanced funnel plot was used to differentiate asymmetry.

## Results

### Study selection

**Fig. 1:**
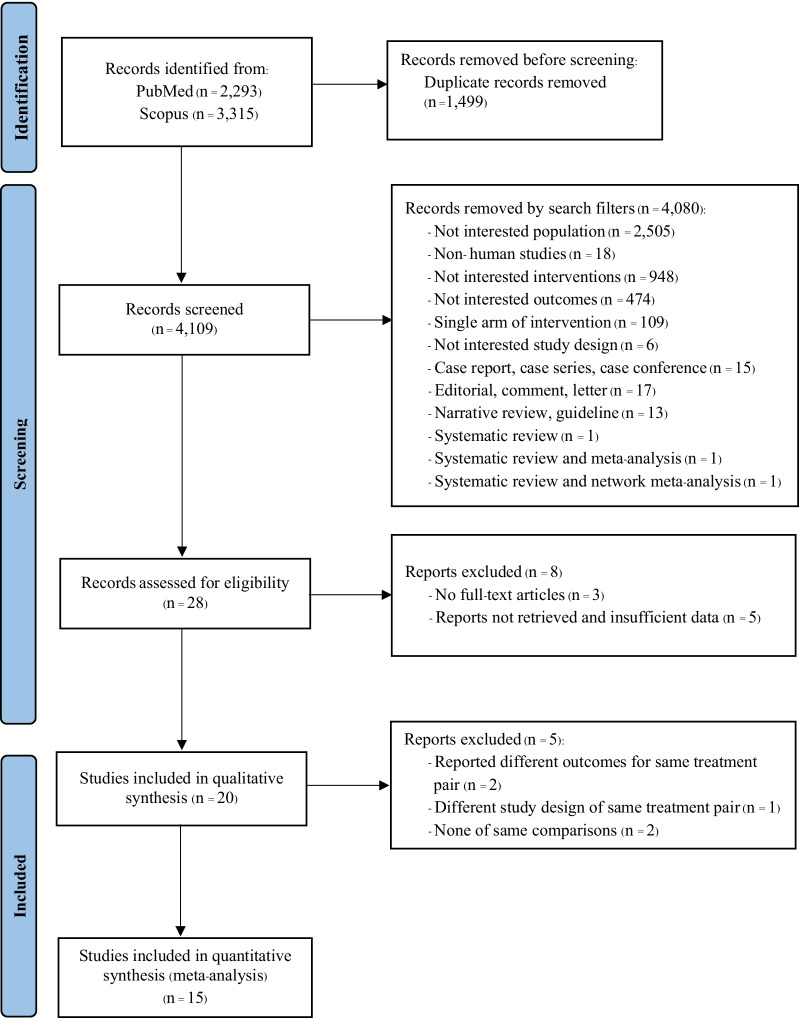
PRISMA flow diagram of study selection process

The study selection process is demonstrated in Fig. 1. We searched 5608 articles: 2293 articles from PubMed and 3315 articles from Scopus, 1499 duplicates were removed, and the remaining were screened by titles and abstracts. Eight articles were excluded due to full-text unavailability and insufficient data for pooling. Twenty studies were eligible for systematic review [7, 9–13, 20, 21, 23, 27–37]. Five studies reported different interventions [28, 37], outcomes [21, 27], and different RCT design for the same treatment pairwise [30]. Finally, 15 studies were included in the meta-analysis [7, 9–13, 20, 23, 29, 31–36].

### Characteristics of the reviewed studies

Twenty studies were systematically reviewed (16 parallel design [7, 9–13, 20, 23, 27, 30–34, 36, 37] and 4 crossover design) [29, 33–35] as shown in Table 2. There were 716 participants, 2–16 years of age, reported mean age of 6 years and 5 months (SD 1 year and 7 months) [9–13, 20, 21, 27–31, 33–37], and 60.9% were male [9–13, 21, 27–37]. Topographic distribution of motor signs was diplegia (76.7%), hemiplegia (22.6%), quadriplegia (0.6%), and triplegia (0.1%) [7, 9–13, 21, 23, 27, 30–32, 34, 35, 37]. Of 7 studies, GMFCS levels I-III were 34.3%, 46.9%, and 18.8%, respectively [21, 29–32, 36, 37]. An average baseline of ankle dorsiflexion was 8 degrees with/without specific knee extension [9–11, 20, 27, 31, 32, 35, 36]. An average follow-up period was 15.6 weeks, ranging from 4 to 48 weeks.

**Table 2:** Characteristics of included studies

First author (country)	RCT design	Sample size (*n*)	Mean age (years)	Gender (*n*)	Topographic distribution (*n*)	GMFCS I/II/III (*n*)	Baseline PROM of ankle DF with KE (mean of degrees)
Baker et al. [27] (Northern Ireland)	Parallel	125	5.34	M = 67 F = 58	Diplegia = 125	N.A	10.38
Bottos et al. [28] (Italy)	Parallel	10	6.4	M = 7 M = 3	N.A	N.A	N.A
Buckon et al. [29] (USA)	Crossover	16	8.4	M = 10 F = 6	N.A	I = 4/II = 12	N.A
Corry et al. [9] (Northern Ireland)	Parallel	20	4.6	N.A	Hemiplegia = 8 Diplegia = 11 Quadriplegia = 1	N.A	− 1.25
Dalvand et al. [30] (Iran)	Parallel	30	6.03	M = 13 F = 17	Diplegia = 30	I = 12/II = 13/III = 5	N.A
Dursun et al. [13] (Turkey)	Parallel	35	9	M = 28 F = 7	Hemiplegia = 6 Diplegia = 29	N.A	N.A
Dursun et al. [31] (Turkey)	Parallel	51	6.4	M = 32 F = 29	Hemiplegia = 14 Diplegia = 37	I = 11/II = 25/III = 15	10.33
El-Etribi et al. [20] (Eqypt)	Parallel	40	3.6	N.A	N.A	N.A	(17.3)
Flett et al. [10] (Australia)	Parallel	18	3.7	M = 11 F = 7	Hemiplegia = 5 Diplegia = 10 Triplegia = 1 Quadriplegia = 2	N.A	7.04
Hayek et al. [32] (Israel)	Parallel	20	3.9	M = 11 F = 9	Hemiplegia = 10 Diplegia = 10	I = 12/II = 5/III = 3	5.8
Koman et al. [7] ( USA)	Parallel	12	4-11^a^	N.A	Hemiplegia = 4 Diplegia = 8	N.A	N.A
Koman et al. [23] ( USA)	Parallel	114	2–16 ^a^	N.A	Hemiplegia = 32 Diplegia = 182	N.A	N.A
Mass et al. [21] (Netherlands)	Parallel	19	8.89	M = 12 F = 7	Hemiplegia = 9 Diplegia = 10	I = 10/II = 7/III = 2	N.A
Radtka et al. [33] ( USA)	Crossover	10	6.5	M = 6 F = 4	Hemiplegia = 4 Diplegia = 6	N.A	≥ 5*
Radtka et al. [34] ( USA)	Crossover	12	7.5	M = 6 F = 6	N.A	N.A	≥ 5*
Rethlefsen et al. [35] ( USA)	Crossover	21	9.1	M = 13 F = 8	Diplegia = 21	N.A	≥ 5*
Sutherland et al. [12] ( USA)	Parallel	20	6.1	M = 16 F = 4	Hemiplegia = 10 Diplegia = 9 Quadriplegia = 1	N.A	≥ 0
Ubhi et al. [11] (England)	Parallel	40	7.43	M = 23 F = 17	Hemiplegia = 12 Diplegia = 28	N.A	− 17.51
Xu et al. [36] ( China)	Parallel	65	4.6	M = 44 F = 21	N.A	I = 24/II = 41	− 8.2
Yigitoglu et al. [37] (Turkey)	Parallel	38	6.3	M = 19 F = 19	Diplegia = 38	I = 9/II = 9/III = 20	N.A

a Age range; N.A., not available; PROM, passive range of motion; DF, dorsiflexion; and KE, knee extension

*A minimum five degrees of PROM

### Intervention and outcome measure

Twenty RCTs were identified as shown in Table 3 comprising 6 treatment pairs: 5 BoNT-A versus placebo [7, 11, 12, 23, 27], 3 BoNT-A plus physical therapy versus physiotherapy [13, 20, 36], 2 BoNT-A versus casting [9, 10], 3 BoNT-A plus casting versus BoNT-A [28, 31, 32], 1 BoNT-A plus physiotherapy versus BoNT-A alone [37], and 6 orthosis versus control [21, 29, 30, 33–35]. The outcomes were reported as number of gait improvement by PRS and VGA, composite scores assessed by PRS, Observational Gait Scale, or CGAS, ankle dorsiflexion during stance from 3-dimensional gait analysis, passive ankle dorsiflexion, and GMFM dimensions D and E.

**Table 3:** Summary of interventions and outcome measurements

Study	Intervention	Additional treatment	Follow-up (weeks)	Outcome measures
Baker et al. [27]	1. Placebo 2. Dysport;10–30 IU/Kg	Conventional PT and orthosis	16	- Mean of PROM of ankle DF (degrees) - Mean score of GMFM dimensions D and E (points)
Bottos et al. [28]	1. Dysport + AFO 2. Dysport + Casting - Dysport15-20 IU/Kg for both groups	Stretching, exercise, standing and gait training and provided AFO after BoNT-A	16	- Peak DF at stance (degrees)
Buckon et al. [29]	1. Control; no AFO 2. Hinged AFO, solid AFO, PLS; 6–12 h/day	No	12	- The 3D gait analysis data; kinematic (degrees) - Mean of PROM of ankle DF (degrees) - Mean score of GMFM dimensions D and E (points)
Corry et al. [9]	1. Casting; frequency of casting depended on clinical response 2. BoNT-A; 6–8 IU/Kg	No	12	- Mean composite scores of PRS (4 subscales, total 10 points/limb) - Mean of PROM of ankle DF (degrees) - The 3D gait analysis data; Mean range of ankle DF at initial contact, peak DF at stance, peak PF (degrees)
Dalvand et al. [30]	1. Control 2. Hinged AFO, solid AFO; applied after casting	NDT 3 months (3 sessions per week, 1 h daily)	12	- Mean difference of mean score of GMFM dimension D and E (points)
Dursun et al.[13]	1. PT 2. BoNT-A + PT - injected into the gastrocsoleus and tibialis posterior including hamstring and hip adductor 8–10 U/Kg	PT; Stretching, strengthening exercise, coordination training, training in daily activities	4	- Mean composite scores of CGAS (points)
Dursun et al. [31]	1. BoNT-A 2. BoNT-A + Casting injected Dysport 10–40 IU/Kg into gastrocsoleus, Casting × 3 times/week	PT (improve functional mobility, stretching) and OT; 1 h/session, 5 sessions/week	12	- Mean composite scores of OGS (points) - Mean of PROM of ankle DF (degrees)
El-Etribi et al. [20]	1. PT 2. BoNT-A + physiotherapy injected BoNT-A 3 U/Kg for hemiplegia and 6 U/Kg for diplegia into gastrocnemius	Stretching, strengthening exercise 1–1.5 h/session, 3 days/weeks	12	- Mean composite scores of PRS (6 subscales, total 14 points/limb) - Mean of PROM of ankle DF (degrees)
Flett et al. [10]	1. Casting; lasted for 4 weeks; reapplied at 2 weeks 2. BoNT-A; 4–8 U/Kg injected into gastrocsoleus	Night plaster in both groups	48	- Mean composite scores of PRS (2 subscales, total 7 points/limb) - Mean of PROM of ankle DF (degrees) - Mean score of GMFM dimension D,E (points)
Hayek et al. [32]	1. BoNT-A 2. BoNT-A + Casting injected BoNT-A into gastrocnemius (total dose of 20 U/Kg) retained casting at 2 weeks for 4 months	- Conventional PT 3 times/week - Brace	48	- Mean composite scores of OGS (points) - Mean of PROM of ankle DF + KE (degrees) - Mean of AROM of ankle DF (degrees) - Mean score of GMFM dimension D,E (points)
Koman et al. [7]	1. Placebo 2. BoNT-A; injected into medial and lateral gastrocnemius for 1 U/Kg of hemiplegia, 2 U/Kg of diplegia	Conventional PT	6	- Number of improvements of PRS (6 subscales, total 14 points/limb)
Koman et al. [23]	1. Placebo 2. BoNT-A; injected into medial and lateral gastrocnemius for 4 U/Kg of hemiplegia, 8 U/Kg of diplegia	Conventional PT	8	- Number of improvements of PRS (6 subscales, total 14 points/limb)
Mass et al. [21]	1. Control; No KAFO at night 2. KAFO for at least 6 h/night	Physical therapy; gait and standing training	48	- Mean of PROM of ankle DF (degrees)
Radtka et al. [33]	1. Control; no AFO 2. Orthosis; solid AFO	8 subjects received PT	4	- Mean range of ankle DF at initial contact and mid-stance (degrees)
Radtka et al. [34]	1. Control; no AFO 3. Orthosis; solid and hinged AFO	Preventing	4	- Mean range of ankle DF at initial contact, mid-stance, terminal stance (degrees)
Rethlefsen et al. [35]	1. Control 2. Orthosis; fixed AFO, articulated AFO	None	6	- The 3D gait analysis data: mean of ankle DF at initial and terminal stance (degrees)
Sutherland et al. [12]	1. Placebo 2. BoNT-A; injected into gastrocnemius for 4 U/Kg of hemiplegia, 4 U/Kg × 2 times for diplegia	None	8	- Number improvement of ankle DF at initial contact (degrees) - Number of improvements of VGA (graded 0–3 score, points) -Mean difference of PROM of ankle DF (degrees)
Ubhi et al. [11]	1. Placebo 2. BoNT-A; injected Dysport 15 U/Kg for hemiplegia, 25 U/Kg for diplegia at gastrocsoleus *3 cases were injected at hamstrings	Conventional PT with orthosis > 3 months before receive intervention	12	- Number of gait improvements of VGA (graded 0–4 score, points) - Number of improvement of GMFM dimension E (points)
Xu et al. [36]	1. PT 2. BoNT-A + PT **-** BoNT-A injected to ankle plantar flexors, 3 U/Kg for hemiplegia, 10 U/Kg for diplegia - PT in both groups; orthosis, NDT, stretching, strength and coordination training and task-specific training, and electrical stimulation (ES) 1–1.5 h/session, 5 days/week for 2 weeks	None	12	- Mean of PROM of ankle DF (degrees) - Mean score of GMFM dimension E (points)
Yigitoglu et al. [37]	1. BoNT- A 2. BoNT-A + electrical stimulation - ES applied to the gastrocnemius muscle for 20 min/1 time, for 10 days - BoNT-A10 U/Kg applied to the gastrocnemius and soleus muscles and home-based exercise programs for both groups	None	12	- Median of score of GMFM dimension E (points)

BoNT-A, Botulinum toxin A; PROM, passive range of motion; DF, dorsiflexion; AFO, ankle–foot orthosis; KAFO, knee–ankle–foot orthosis; PLS, posterior leaf spring; NDT, neurodevelopment therapy; ES, electrical stimulation; PT, physical therapy; OT, occupational therapy; PRS, Physician’s Rating Scale; VGA, Video Gait Analysis; OGS, Observational Gait Scale; CGAS, Clinical Gait Assessment Score, PROM, passive range of motion; AROM, active range of motion; and GMFM, the Gross Motor Function Measure

### Quality assessment

Thirty-five percent of the studies were at low risk, 55% with some concerns, and 10% with high risk of bias as shown in Table 4. Most studies (60%) did not specify randomization process, allocation sequence, concealment [7, 9, 13, 20, 23, 28, 29, 31, 32, 34–36], and one without ascertainment on the awareness of outcome assessors [19]. Five studies evaluated orthosis [29, 30, 33–35] did not provide randomization and concealment methods.

**Table 4:** Risk-of-bias assessment of included RCTs

Study	Randomization process	Deviations from intended intervention	Missing outcome data	Measurement of the outcome	Selection of the reported result	Overall
Baker et al. [27]	Low	Low	Low	Low	Low	Low
Bottos et al. [28]	Some concerns	Low	Low	Low	Low	Some concerns
Buckon et al. [29]	Some concerns	Low	Low	Low	Low	Some concerns
Corry et al. [9]	Some concerns	Low	Low	Low	Low	Some concerns
Dalvand et al. [30]	Low	Low	Low	Low	Low	Low
Dursun et al. [13]	Some concerns	Low	Low	Low	Low	Some concerns
Dursun et al. [31]	Some concerns	Low	Low	Low	Low	Some concerns
El-Etribi et al. [20]	Some concerns	Low	Low	High	Low	High
Flett et al. [10]	Low	Low	Low	Low	Low	Low
Hayek et al. [32]	Some concerns	Low	Low	Low	Low	Some concerns
Koman et al. [7]	Some concerns	Low	Low	Low	Low	Some concerns
Koman et al.[23]	Some concerns	Low	Low	Low	Low	Some concerns
Maas et al. [21]	Low	Low	Low	Low	Low	Low
Radtka et al. [33]	High	Low	Low	Low	Low	High
Radtka et al. [34]	Some concerns	Low	Low	Low	Low	Some concerns
Rethlefsen et al. [35]	Some concerns	Low	Low	Low	Low	Some concerns
Sutherland et al. [12]	Low	Low	Low	Low	Low	Low
Ubhi et al. [11]	Low	Low	Low	Low	Low	Low
Xu et al. [36]	Some concerns	Low	Low	Low	Low	Some concerns
Yiğitoğlu et al. [37]	Low	Low	Low	Low	Low	Low

### Meta-analysis of intervention studies

Five studies [21, 27, 28, 30, 37] were excluded due to different study designs, interventions, and outcomes leaving 15 studies [7, 9–12, 20, 23, 29, 31–36] for the meta-analysis. Network meta-analysis could not be done due to the lack of a common comparator.

#### Gait improvement by visual observational gait analysis

**Fig. 2:**
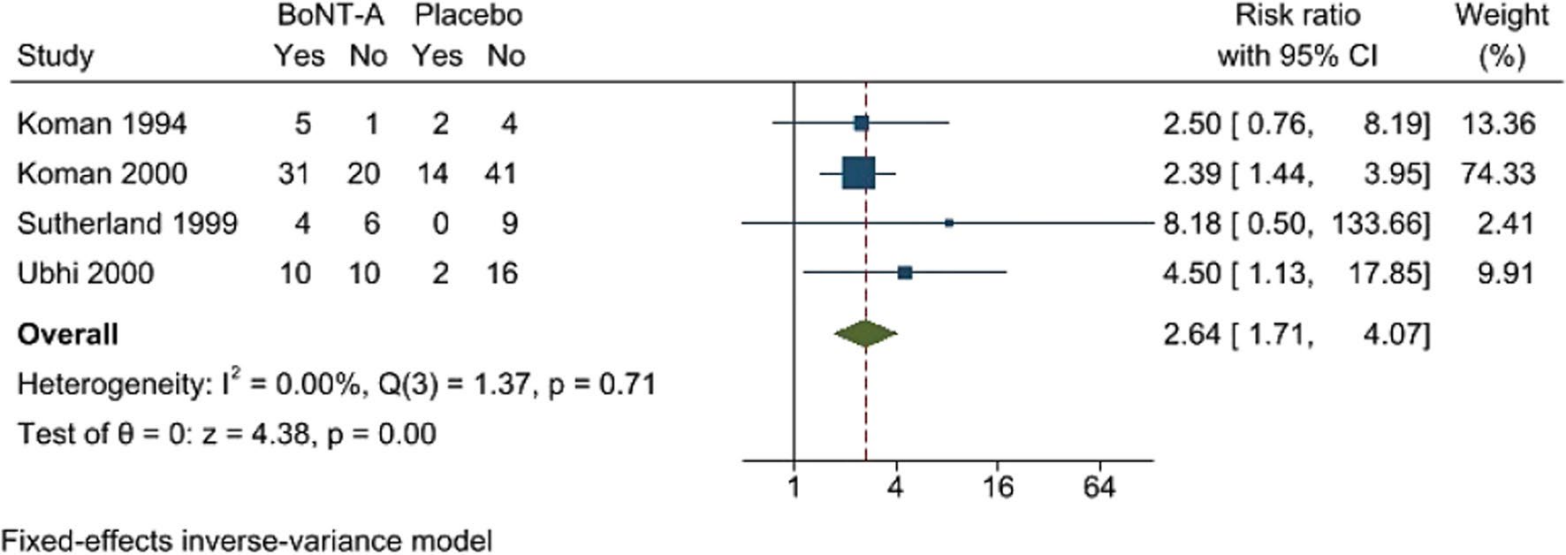
Forest plot showing meta-analysis of Botulinum toxin A (BoNT-A) versus placebo on number of gait improvement. Gait improvement was determined by at least 2 scores of Physician’s Rating Scale [7, 23] or at least of 1 point of Video Gait Analysis improvement [11, 12]

**Fig. 3:**
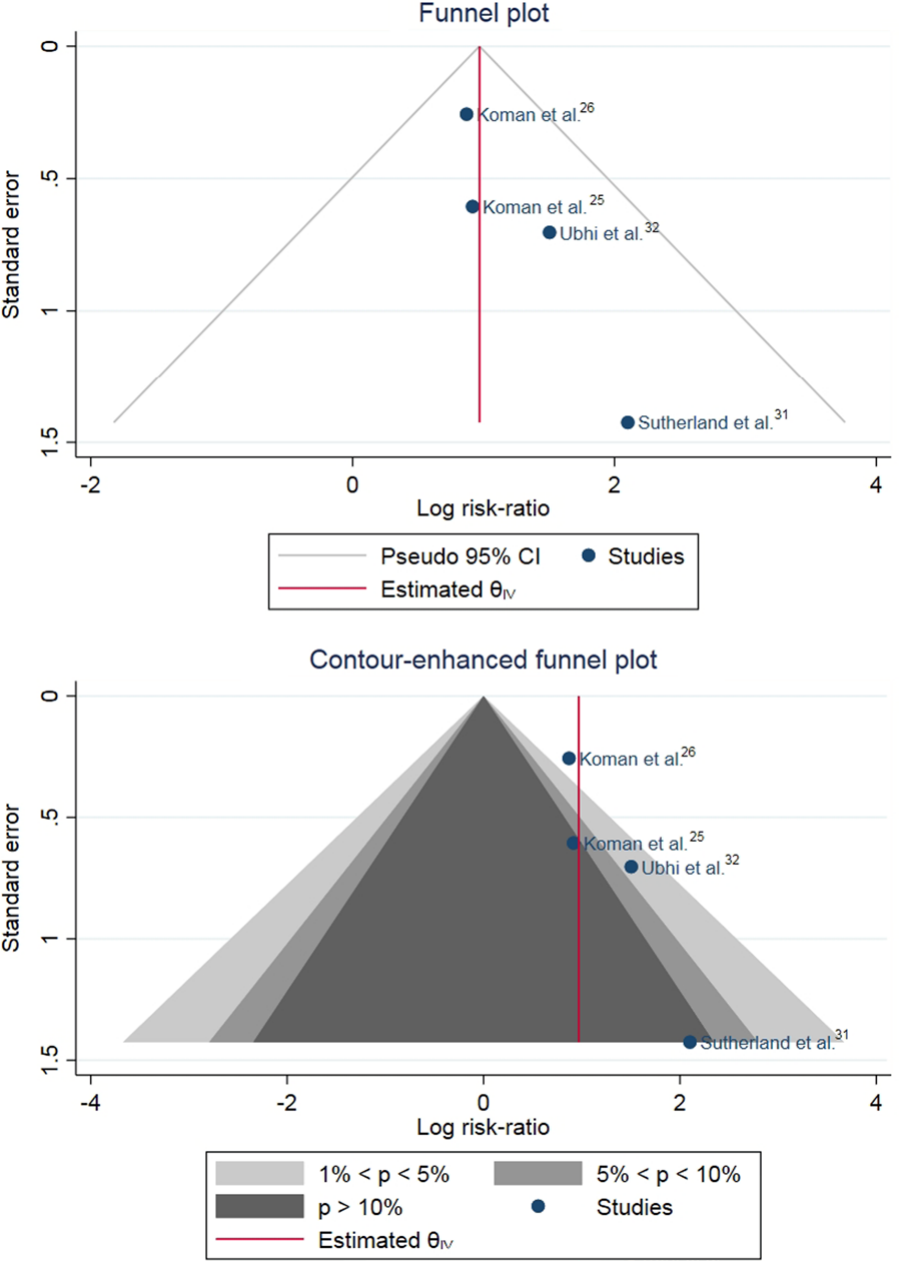
A funnel plot of the comparison of botulinum toxin A (BoNT-A) versus placebo showed asymmetry of the plot. A contour-enhanced funnel plot demonstrated that missing studies were in the area of non-significance indicating a publication bias

After categorized as clinical improvement vs no improvement, both scales (PRS, VGA) can be pooled for the analysis. BoNT-A had significantly higher numbers of gait improvement by PRS and VGA at 6–12 weeks comparing to the placebo (RR 2.64; 95%CI 1.71, 4.07, no heterogeneity) [7, 11, 11, 23] (Fig. 2). A funnel plot was asymmetric, and a contour-enhanced funnel plot showed missing published studies in a non-significant area indicating a publication bias (Fig. 3).

**Fig. 4:**
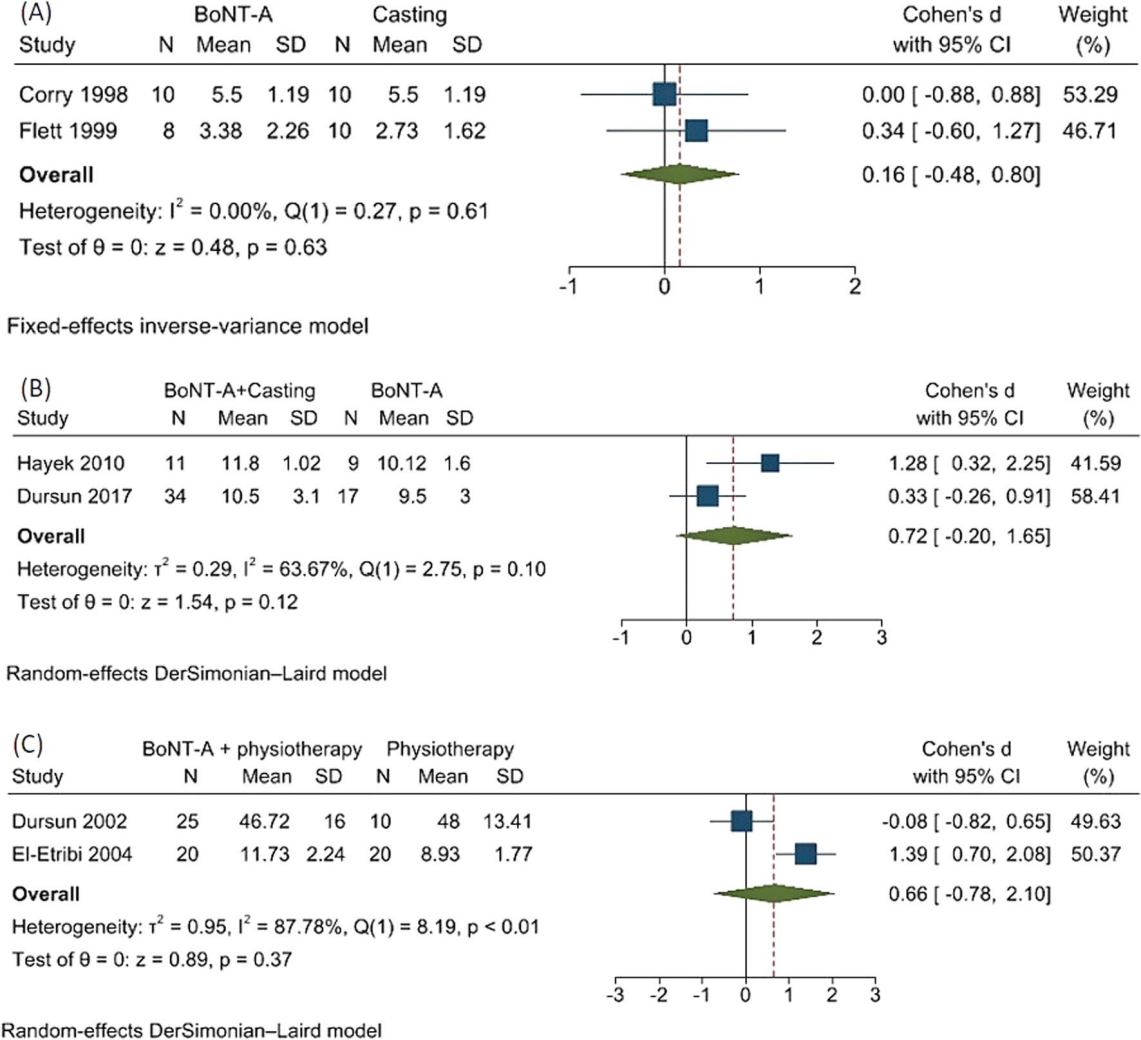
Forest plots showing meta-analysis for composite scores of **A**. botulinum toxin A (BoNT-A) versus casting evaluated by composite scores of Physician’s Rating Scale [9, 10], **B**. BoNT-A plus casting versus BoNT-A alone assessed by composite scores of Observational Gait Scale [31, 32], and **C**. BoNT-A plus physical therapy versus physical therapy indicated by composite scores of Clinical Gait Assessment Score [13, 20]

BoNT-A showed no significant differences of the PRS composite scores at 12 and 16 weeks comparing to casting (SMD 0.16; 95%CI − 0.48,0.80, no heterogeneity) [9, 10] (Fig. 4A). BoNT-A plus casting demonstrated no significant differences of the Observational Gait Scale composite scores at 12 and 16 weeks when compared to BoNT-A alone (SMD 0.72; 95%CI − 0.20,1.65, moderate heterogeneity, *I*^2^ = 63.67%, *Q* = 2.75, and *p* = 0.10) [31, 32] (Fig. 4B). In addition, the combination of BoNT-A with physical therapy had non-statistically different PRS and CGAS composite scores at 4 and 12 weeks from physical therapy (SMD 0.66; 95%CI − 0.78,2.10), high heterogeneity, *I*^2^ = 87.78%, *Q* = 8.19, and *p* < 0.01) [13, 20] (Fig. 4C). The high heterogeneity may be from different gait assessment scales.

#### Three dimensional gait analysis

**Fig. 5:**
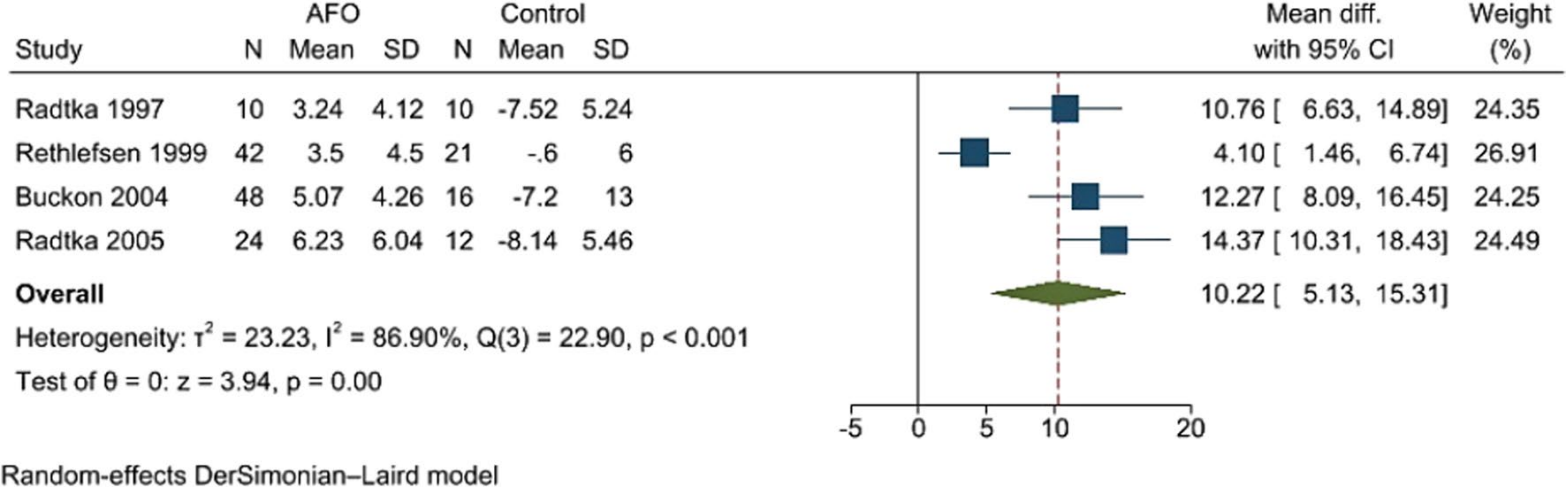
Forest plot showed meta-analysis of the efficacy of the ankle–foot orthosis (AFO) versus control on ankle dorsiflexion at initial contact

**Fig. 6:**
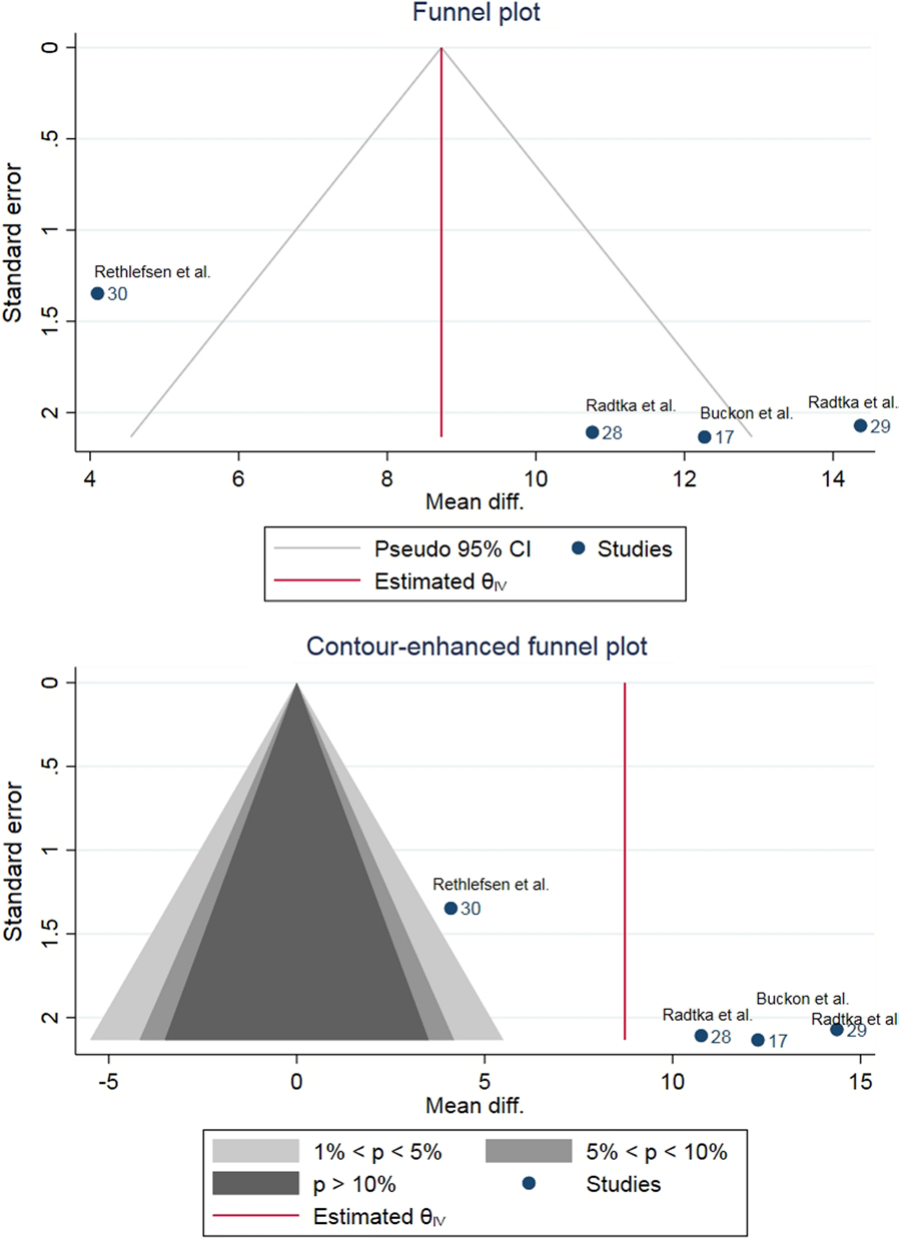
A funnel plot and a contour-enhanced funnel plot of the comparison between orthosis and control on ankle dorsiflexion showed asymmetry. Missing studies were broadly in the area of statistical significance (no shading) which indicated the influences from other factors rather than a publication bias

Ankle–foot orthosis significantly increased ankle dorsiflexion at initial contact comparing to control, i.e., shoes or barefoot (USMD 10.22 degrees; 95%CI 5.13, 15.31, high heterogeneity, *I*^2^ = 86.9%, *Q* = 22.9, and *p* < 0.001 (Fig. 5). A funnel plot and a contour-enhanced funnel plot were asymmetric, which indicated the influences from other factors rather than a publication bias [26] (Fig. 6).

#### Passive range of ankle dorsiflexion at knee extension

**Fig. 7:**
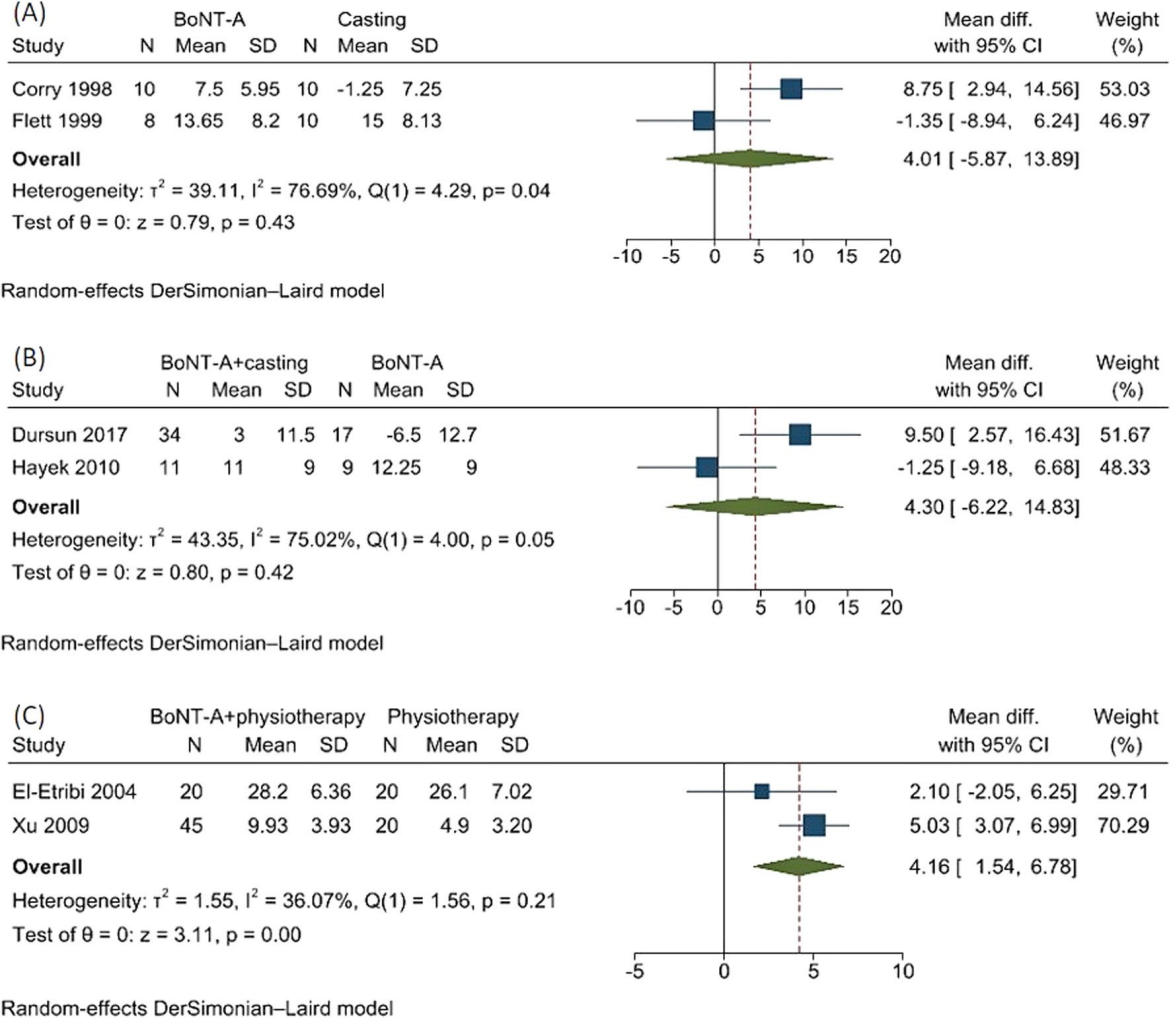
Forest plot showing meta-analysis for passive ankle dorsiflexion with knee extension of **A** botulinum toxin A (BoNT-A) versus casting alone, **B** BoNT-A plus casting versus BoNT-A alone, and **C** BoNT-A plus physical therapy versus physical therapy alone

There were 3 treatments comparing the passive range of ankle dorsiflexion at knee extension. BoNT-A versus casting showed non-significant difference (USMD 4.01 degrees; 95% CI − 5.87,13.89, high heterogeneity, *I*^2^ = 76.69%, *Q* = 4.29, and *p* = 0.04)[9, 10] (Fig. 7A). BoNT-A plus casting versus BoNT-A alone demonstrated non-significant difference (USMD 4.30 degrees; 95% CI − 6.22, 14.83, moderate heterogeneity, *I*^2^ = 75.02; *Q* = 4.00, and *p* = 0.05) [31, 32] (Fig. 7B). BoNT-A plus physical therapy versus physical therapy yielded a statistically significant difference in ankle dorsiflexion (USMD 4.16 degrees; 95%CI 1.54, 6.78, moderate heterogeneity, *I*^2^ = 36.07%, *Q* = 1.56, and *p* = 0.21) [20, 36] (Fig. 7C).

#### Gross Motor Function Measure (GMFM); dimensions D and E

Five studies reported GMFM dimension D and E [10, 27, 29, 30, 32], whereas three studies assessed only dimension E [11, 36, 37]. All studies reported various comparisons and could not be pooled in the meta-analysis. Individual studies showed no statistically significant for BoNT-A versus placebo [11, 27], BoNT-A plus casting versus BoNT-A alone [32], BoNT-A versus casting [10], BoNT-A plus physiotherapy versus physiotherapy alone [36], and BoNT-A plus physiotherapy versus BoNT-A alone [37]. Both hinged and solid AFO improved GMFM dimension E [29], whereas only hinged AFO provided better GMFM dimensions D and E than controls [30].

The summary of estimated treatment effects for all comparisons is provided in Table 5. Average BoNT-A dosage from included studies was 3 U/Kg body weight [7, 9–11, 13, 20, 23, 31, 32].

**Table 5:** Summary of estimated treatment effect of the included studies

Outcome measure	Treatment comparisons	Pooled effect size
1. Number of gait improvement	BoNT-A versus placebo [7, 11, 12, 23]	RR (95% CI); 2.64 (1.71, 4.07)
2. Composite score of gait improvement	a. BoNT-A versus casting [9, 10]	SMD (95% CI); 0.16 (− 0.48, 0.8)
	c. BoNT-A + casting versus BoNT-A [31, 32] b. BoNT-A + physical therapy versus physical therapy [13, 20]	SMD (95% CI); 0.72 (− 0.2, 1.65) SMD (95% CI); 0.66 (− 0.78, 2.1)
3. Ankle dorsiflexion at initial contact	Orthosis versus control [29, 33–35]	USMD (95% CI); 10.22 (5.13, 15.31)
4. Passive range of ankle dorsiflexion with knee extension	a. BoNT-A versus casting [9, 10]	USMD (95% CI); 4.01 (− 5.87, 13.89)
	b. BoNT-A + casting versus BoNT-A [31, 32] c. BoNT-A + physical therapy versus physical therapy [20, 36]	USMD (95%CI); 0.39 (− 0.52, 1.3) USMD (95% CI); 4.16 (1.54, 6.78)

Botulinum toxin A, BoNT-A; RR, risk ratio; SMD, standardized mean difference; USMD, unstandardized mean difference; and CI, confidence interval

## Discussion

This systematic review with meta-analysis was conducted to assess the efficacy of conservative treatments, i.e., BoNT-A, physiotherapy, casting, and AFO in promoting quality of gait in children with spastic cerebral palsy presenting with equinus foot. According to the studies, BoNT-A improved gait patterns, assessed by PRS and VGA more than placebo with/without conventional therapy. No significant differences in gait improvement were detected among other pairings included BoNT-A versus casting, BoNT-A plus casting versus BoNT-A alone, and BoNT-A plus physiotherapy versus physiotherapy alone. Interestingly, the BoNT-A combining with physiotherapy could significantly maximize passive ankle dorsiflexion by 4 degrees comparing to physiotherapy alone. Moreover, the AFO improved gait pattern by increasing the ankle dorsiflexion during initial contact as well as the gross motor function comparing to non-AFO group. The results from this study would provide proper clinical decision to conservatively manage equinus deformity.

Our finding reaffirms that the BoNT-A was effective for improving gait and its effect with physical therapy in enhancing passive ankle dorsiflexion at knee extension comparing to physical therapy alone. Although our research and the previous meta-analysis [17] included the same studies [7, 11, 12, 23], the previous review showed high effect size with bias estimation from Peto odds ratio at 3.99; 95%CI 1.89, 8.44, which is not recommended as a default method for meta-analysis due to possible over-estimation of effect size[17]. The BoNT-A may take at least 8 weeks for gait improvement efficacy and then can be clinically apparent at 12–16 weeks [23]. Most of included studies followed up to 12–16 weeks and focused on spastic cerebral palsy in early to middle childhood. During this age period, it is the optimal time to start BoNT-A due to flexible gait patterns and gross motor function [38]. The BoNT-A dosage from our review is 3 U/Kg/body weight, which is quite compatible with the common use of 4–8 U/kg/body [38], and multilevel BoNT-A 2 to 29 U/kg/body weight [38] for severe spasticity with multiple contractures [39]. In addition, our study points out that the BoNT-A plus physiotherapy could significantly increase the passive ankle dorsiflexion when compared to the physiotherapy alone, but the previous study did not estimate this effect.

Casting immobilized and lengthened muscle position by reducing spasticity and also enhancing gait function and ankle movement [40]. With regard to the previous systematic review [41], the BoNT-A showed non-significant gait improvement and passive ankle dorsiflexion with knee extension when combined or compared with casting. We also deepened the analysis by including more outcomes such as mean composite scores of PRS, Observational Gait Scale, and CGAS, and ankle dorsiflexion at initial contact. The results still showed insignificant difference. The possible explanation may be from heterogeneity caused by gait assessment scales, small number of studies, and baseline passive ankle dorsiflexion. On the contrary, the network meta-analysis found that BoNT-A significantly improved passive ankle dorsiflexion with knee extension at 3 months compared to BoNT-A plus casting [18]. These inconsistent results may be due to different inclusion criteria, i.e., study designs and interventions (immediate/delayed casting).

The AFO was known to enhance ankle dorsiflexion during walking. Our results confirmed its ability to increase ankle dorsiflexion at initial contact (USMD = 10.22, 95%CI 5.13, 15.31 converted to SMD = 1.62; 95% CI 0.82, 2.43). The previous meta-analysis recruited both observational studies and RCTs with the SMD = 1.34 and 95%CI from 1.13, 1.56 [19]. However, pooling different study designs may increase the risk of biases from the high heterogeneity of population and confounding factors [42]. Our study tried to explore the differences between two included studies comparing AFO versus control. Buckon et al. summarized a significant different GMFM dimension E between both hinged and solid AFO versus control [29], whereas the other reported that only hinged AFO provided significantly different GMFM dimensions D and E from control [30]. The conflicting results may come from different study designs: crossover (controlled within subjects) [29] and quasi-experimental study (not randomly assigned) [30]. Moreover, a hinged AFO allows free dorsiflexion but blocks plantar flexion at 0° [5]. We, therefore, performed subgroup analysis to compare between hinged and solid AFO [29, 34, 35]. Hinged design increased ankle dorsiflexion at initial contact more than a solid type without significant difference (USMD = 0.37; 95%CI − 1.48, 2.22).

The strengths of our study are the inclusion of all conservative treatments, performing meta-analysis, and estimated overall gait outcomes. We employed a comprehensive search strategy without limiting to only English language; followed the PRISMA guideline; and retrieved only RCTs and good quality assessment (90% of low risk/some concern). However, limitations are various placebo and control, as well as no BoNT-A vs AFO precluded common comparators between AFO/physical therapy and other treatments to conduct an indirect comparison. Publication bias was found among BoNT-A vs placebo comparisons [7, 11, 12, 23]. Most published RCTs investigated non-specific GMFCS [7, 9–13, 20, 23, 28, 33–35, 43]; small number of studies focused on pre-treatment ankle passive motion [9–11, 20, 31–36, 43]; and small sample size [7, 10, 21, 28, 29, 33, 34] leading to inconsistency and weakness of evidences. The characteristics of equinus were not clearly identified. Therefore, we determined dynamic equinus at ankle dorsiflexion less than 10 degrees [44] instead of unreliable passive range of motion [10]. Furthermore, the outcomes were evaluated at a short period of 3–4 months, but it was adequate to detect gait improvement [23].

In clinical practice for spastic equinus deformity, BoNT-A or casting may be chosen according to availability or affordability. AFO is the other option to enhance ankle dorsiflexion at initial contact and GMFM. Further randomized controlled trials comparing gait improvement and ankle dorsiflexion between AFO and BoNT-A, a common comparator, would facilitate a network meta-analysis to find the best treatment and fill the gap of knowledge.

## Conclusion

BoNT-A, casting, and AFO could be recommended for a young ambulatory/partially ambulatory cerebral palsy with dynamic equinus deformity. Either BoNT-A or casting contributes to gait improvement by visual observational gait analysis and passive ankle dorsiflexion. Moreover, BoNT-A provides additional passive ankle dorsiflexion with knee extension to physiotherapy alone. AFO would be useful for ankle dorsiflexion at initial contact and gross motor function.

## Supplementary Information

**Additional file 1.** Search terms and search strategy.

## Data Availability

The datasets used and/or analyzed during the current study are available from the corresponding author on reasonable request.

## Abbreviations

RCT: Randomized controlled trial
BoNT-A: Botulinum toxin A
RR: Risk ratio
USMD: Unstandardized mean difference
CI: Confidence interval
PRS: Physician’s Rating Scale
GMFM: Gross Motor Function Measure
VGA: Video Gait Analysis
AFO: Ankle–foot orthosis
PRISMA: Preferred Reporting Items for Systematic Reviews and Meta-Analyses
GMFCS: Gross Motor Function Classification System
CGAS: Clinical Gait Assessment Score
RoB 2: A revised Cochrane risk-of-bias tool for randomized trials
SMD: Standardized mean difference
