# Supplementary material for: Efficacy of conservative treatment for spastic cerebral palsy children with equinus gait: a systematic review and meta-analysis
Source: J Orthop Surg Res. 2022 Sep 8;17:411. doi: 10.1186/s13018-022-03301-3 (PMC9461190; doi:10.1186/s13018-022-03301-3)
Supplement: Supplementary file 1 — Additional file 1. Search terms and search strategy. [file 13018_2022_3301_MOESM1_ESM.docx]

**Search terms and search strategy**

Search terms for P, I/C and O were as follows:

| **Domain** | **Search term** | **Covered term** |
| --- | --- | --- |
| **Patient** | | |
| P1**:** cerebral palsy | “cerebral palsy” | - “Cerebral Palsy”[Mesh] - “spastic cerebral palsy” |
| P2**:** Equinus deformity | equin* | - “EquinusDeformity”[Mesh] - equinus |
|  | tiptoe*  toe* | - tiptoeing - “toe walking” |
| **Intervention** | | |
| I1: Botulinum toxin type a | Botulinum*  Botox  “BTX”  BoTN-A  Dysport | - “Botulinum Toxin” - “Botulinum Toxins”[Mesh] - “botulinum a toxin” - “Botulinum toxin A” - “botulinum toxin type a” |
| I2: Cast | Casting | - “Casts, Surgical”[Mesh]   “serial casting” |
|  | Casts |  |
|  | Cast |  |
| I3: Orthosis | Orthosis  Orthoses  Orthotic | - Orthotics - “Orthotic Devices”[Mesh] |
|  | Splint | - “Splints”[Mesh] |
|  | Brace | - Bracing |
| I4: Physiotherapy | Physiotherapy | - "physical therapy modalities"[MeSH Terms] - "physiotherapies" |
|  | “Physical therapy” |  |

Search terms for P, I/C and O were as follows (continue):

| **Domain** | **Search term** | **Covered term** |
| --- | --- | --- |
| **Outcome** | | |
| O1: Gait | “Walk” | - "Walking"[Mesh] |
|  | Gait | - “gait analysis” - “gait assessment” - “gait improvement” - “gait parameter” - “Gait”[Mesh] |
|  | Kinematics |  |
| O2: Range of motion | “Range of motion” | - "Range of Motion, Articular”[Mesh]” - “Passive range of motion” |
| O3: Gross motor function | “Motor function” | - “Gross motor function” |

**Table 1: Search strategy and results in MEDLINE via PubMed**

| Domain(S) | Search number | Query | Items  found |
| --- | --- | --- | --- |
| P1 | #1 | Search “cerebral palsy” | 30,314 |
| P2 | #2 | Search equin* | 57,988 |
|  | #3 | Search tiptoe* | 341 |
|  | #4 | Search toe* | 17,509 |
| P2 | #5 | Search ((equin* OR tiptoe*)) OR toe*) | 75,403 |
| P1&P2 | #7 | Search (“cerebral palsy”)AND ((equin* OR tiptoe*)) OR toe*) | 692 |
| I1 | #8 | Search botulinum* | 22,085 |
|  | #9 | Search “BTX” | 2,831 |
|  | #10 | Search “BoNT-A” | 2,132 |
|  | #11 | Search Botox | 11,185 |
|  | #12 | Search Dysport | 868 |
|  | #13 | Search ((((botulinum*) OR ("BTX")) OR ("BoNT-A")) OR (Botox)) OR (Dysport) | 27,023 |
| I2 | #14 | Search casting | 40,693 |
|  | #15 | Search casts | 40,693 |
|  | #16 | Search cast | 33,230 |
|  | #17 | Search **((casts) OR (cast)) OR (casting)** | 66,033 |
| I3 | #18 | Search orthosis | 18,217 |
|  | #19 | Search orthoses | 17,713 |
|  | #20 | Search orthotic | 18,788 |
|  | #21 | Search brace | 13,398 |
|  | #22 | Search Splint | 19,854 |
|  | #23 | Search **((((orthosis) OR (orthoses)) OR (orthotic)) OR (brace)) OR (Splint)** | 45,001 |
| I4 | #24 | Search physiotherapy | 212,559 |
|  | #25 | Search “physical therapy” | 97,326 |
|  | #26 | Search (physiotherapy) OR ("physical therapy") | 258,70 |
| I1/I2/I3/I4 | #27 | Search #13 OR #17 OR #23 OR #26 | 303,052 |
| O1 | #28 | Search gait | 72,496 |
|  | #29 | Search walk | 146,318 |
|  | #30 | Search walking | 117,912 |
|  | #31 | Search kinematics | 162,483 |
|  | #32 | Search (((gait OR (walk) OR (walking) OR (Kinematics)) | 320,681 |
| O2 | #32 | Search "range of motion" | 97,521 |
| O3 | #33 | Search “Motor function” | 26,165 |

**Table 1: Search strategy and results in MEDLINE via PubMed (cons.)**

| Domain(S) | Search number | Query | Items  found |
| --- | --- | --- | --- |
| O1/O2/O3 | #34 | Search #32 OR #32 OR #33 | 316,607 |
| P1& I | #35 | Search #1 AND #27 | 2,293 |
| P1& I& O | #36 | Search #1 AND #27 AND #34 | 1,755 |
| P1& P2& I | #43 | Search #7 AND #27 | 308 |
| P1& P2, I, O | #44 | Search #7 AND #27 AND #34 | 225 |
|  |  |  |  |

**Table 2: Search strategy and results in Scopus**

| Domain(S) | Search number | Query | **Item founds** | |  |
| --- | --- | --- | --- | --- | --- |
| P1 | #1 | TITLE-ABS-KEY (“cerebral palsy”) | 43,134 | | |
| P2 | #2 | TITLE-ABS-KEY (equin*) | 71,102 | | |
|  | #3 | TITLE-ABS-KEY (“toe walk*” ) | 465 | | |
|  | #4 | TITLE-ABS-KEY (tiptoe*) | 501 | | |
|  | #5 | TITLE-ABS-KEY #2 OR #3 OR #4 | | 70,933 |  |
| P1&P2 | #6 | ALL #1 AND #5 | | 863 |  |
| I1 | #7 | TITLE-ABS-KEY (Botulinum*) | | 40,429 |  |
|  | #8 | TITLE-ABS-KEY (“BTX-A”) | | 1,238 |  |
|  | #9 | TITLE-ABS-KEY (“BoNT-A”) | | 2,096 |  |
|  | #10 | TITLE-ABS-KEY (Botox) | | 6,180 |  |
|  | #11 | TITLE-ABS-KEY (dysport) | | 2,287 |  |
|  | #12 | TITLE-ABS-KEY #7 OR #8 OR #9 OR #10 OR #11 | | 40,547 |  |
| I2 | #13 | TITLE-ABS-KEY ( cast ) | | 215,838 | |
|  | #14 | TITLE-ABS-KEY ( casting ) | | 180,116 | |
|  | #15 | TITLE-ABS-KEY #13 OR #14 | | 363,934 | |
| I3 | #16 | TITLE-ABS-KEY (orthotics) | | 10,277 |  |
|  | #17 | TITLE-ABS-KEY (orthosis) | | 14,067 |  |
|  | #18 | TITLE-ABS-KEY (orthoses) | | 30,032 |  |
|  | #19 | TITLE-ABS-KEY (brace) | | 25,684 |  |
|  | #20 | TITLE-ABS-KEY (bracing) | | 11,305 |  |
|  | #21 | TITLE-ABS-KEY (splint) | | 20,734 |  |
|  | #22 | TITLE-ABS-KEY #16 OR #17 OR #18 OR #19 OR #20 OR #21 | | 98,225 |  |
| I4 | #23 | TITLE-ABS-KEY (physiotherapy) | | 99,759 |  |
|  | #24 | TITLE-ABS-KEY (“physical therapy”) | | 66,052 |  |
|  | #25 | TITLE-ABS-KEY #23 OR #24 | | 125,007 |  |
| I1/I2/I3/I4 | #26 | TITLE-ABS-KEY #12 OR #15 OR #22 OR #25 | | 624,282 |  |

**Table 2: Search strategy and results in Scopus (cons)**

| Domain(S) | Search number | Query | Items  found |
| --- | --- | --- | --- |
| O1 | #27 | TITLE-ABS-KEY (gait) | 121,683 |
|  | #28 | TITLE-ABS-KEY (walking) | 192,531 |
|  | #29 | TITLE-ABS-KEY (walk) | 125,710 |
|  | #30 | TITLE-ABS-KEY (kinematic) OR TITLE-ABS-KEY (kinematics) | 247,953 |
| O2 | #31 | TITLE-ABS-KEY (“range of motion”) | 101,524 |
| O3 | #32 | TITLE-ABS-KEY (“motor function”) | 84,027 |
| O1/O2/O3 | #33 | TITLE-ABS-KEY #27 OR #28 OR #29 OR #30 OR #31 OR #32 | 653,933 |
| P1&I | #51 | TITLE-ABS-KEY #18 AND #39 | 3,315 |
| P1&I& O | #52 | TITLE-ABS-KEY #18 AND #39 AND #50 | 2,210 |
| P1&P2&& I | #53 | TITLE-ABS-KEY #19 AND #39 | 389 |
| P1&P2&I&O | #54 | TITLE-ABS-KEY #19 AND #39 AND #50 | 282 |

| **Domains** | **PubMed** | **Scopus** |
| --- | --- | --- |
| P1,I, | 2,293 | 3,315 |
| P1,I,O | 1,755 | 2,210 |
| P1&P2,I | 308 | 389 |
| P1&P2,I,O | 225 | 282 |

For finalizes searching, we have decided to use search term of “P1” and “I” for PubMed. While Scopus we will select search term of “P1”, “P2” and “I”
